# Heterogeneity of psychosocial functioning in patients with bipolar disorder: Associations with sociodemographic, clinical, neurocognitive and biochemical variables

**DOI:** 10.3389/fpsyt.2022.900757

**Published:** 2022-09-20

**Authors:** Zhengling Ba, Minhua Chen, Jiulan Lai, Yingtao Liao, Hengying Fang, Dali Lu, Yingjun Zheng, Kunlun Zong, Xiaoling Lin

**Affiliations:** ^1^School of Nursing, Sun Yat-sen University, Guangzhou, China; ^2^Department of Psychiatry, The Third Affiliated Hospital of Sun Yat-Sen University, Guangzhou, China; ^3^Department of Psychiatry, Xiamen Xianyue Hospital, Xiamen, China; ^4^Department of Psychiatry, The Affiliated Brain Hospital of Guangzhou Medical University (Guangzhou Huiai Hospital), Guangzhou, China

**Keywords:** bipolar disorder, psychosocial functioning, neurocognition, biochemical index, cluster analysis

## Abstract

**Objective:**

This study aims to identify the functional heterogeneity in fully or partially remitted patients with bipolar disorder and explore the correlations between psychosocial functioning and sociodemographic, clinical, neurocognitive and biochemical variables.

**Methods:**

One hundred and forty fully or partially remitted patients with bipolar disorder (*BD*) and seventy healthy controls were recruited. The patients were grouped into different profiles based on the Functioning Assessment Short Test (*FAST*) domain scores by hierarchical cluster analysis. The characteristics of subgroups and the correlations between psychosocial functioning and sociodemographic, clinical, neurocognitive and biochemical variables in each cluster were then analyzed.

**Results:**

There were three subgroups in fully or partially remitted patients with BD: the lower functioning group *(LF*), performed global functioning impairments; the moderate functioning group (*MF*), presented selective impairments in functional domains; and the good functioning subgroup (*GF*), performed almost intact functioning. Among the three subgroups, there were differences in FAST domains, sociodemographic variables, clinical variables, some neurocognitive domains and several biochemical indexes.

**Conclusions:**

The study successfully identified three functional subgroups. The characteristics of discrete subgroups and the specific clinical factors, neurocognitive domains and biochemical indexes that are correlated with functional subgroups will allow for making tailored interventions to promote functional recovery and improve the quality of life.

## Introduction

Bipolar disorder (BD) is a common severe and lifelong mental disease characterized by alternating emotional symptoms (1, 2) which cause severe impairment in psychosocial functioning (3). Around 30%-60% patients with BD suffer from psychosocial functioning impairment that seriously affects functional recovery. Psychosocial dysfunction, such as occupational functioning, learning functioning, etc., could reduce occupational competitiveness and quality of life in bipolar disorder (4–7). Psychosocial functioning impairment can be present across different phases in patients with BD, even during remitted phase (8). A 2-year follow-up study showed that although 64% of patients with BD can achieve clinical remission, only 34% of patients achieved functional recovery (9). In recent years, the treatment of patients with BD has begun to pay more attention to functional recovery and prognosis (10), suggesting that functional recovery is as equal important as clinical remission.

Heterogeneity of neurocognition has been reported in schizophrenia at earlier but only a few studies could be found in bipolar disorder (11). Burdick et al. provided empirical evidence on significant heterogeneity of neurocognition in patients with BD, and three different neurocognitive subgroups were found, meanwhile, the demographic and clinical characteristics were different among these subgroups (12), which was consistent with the other study (13). And evidence suggests that heterogeneity of psychosocial functioning in BD might exist, which was based on the same or even better neurocognitive performance as the general population (4, 14). In addition, Sole et al. first confirmed that in euthymic patients with BD also existed three different functional subgroups based on the Functioning Assessment Short Test (*FAST*) domain scores, and three groups have been reported as low functioning group (LF), intermediate functioning group (MF) and good functioning group (GF) (15). Although the above-mentioned studies demonstrated the functional heterogeneity in patients with bipolar disorder, comparatively little is known about it. Moreover, the FAST scale was extensively used to assess the psychosocial functioning of patients with BD, which can comprehensively cover almost all areas of psychosocial functioning (16).

Several sociodemographic, clinical and neurocognitive factors such as age, gender, subthreshold symptoms, age of onset, number of depressive or hypo/manic episodes, psychotic symptoms, chronicity, comorbidities and cognitive reserve have been suggested to influence psychosocial functioning in patients with BD (10, 15, 17–20). Neurocognitive impairment has been demonstrated to be a key predictor of poor functional outcomes (21, 22), which is highly related to functional deficits in patients with BD (23). Especially, the executive function, verbal memory and attention were considered as predictors of good psychosocial functioning (14). In addition, our previous research and other studies have revealed that cognitive reserve plays an important role between neurocognitive functioning and psychosocial functioning (8, 24). Furthermore, the number of episodes, especially more depressive episodes was significantly related to lower functioning (3). In addition, the suicidal ideations and psychotic symptoms in lately episode were correlated with poor quality of life in BD patients (17, 25). And the relationship between suicidal ideations and poor quality of life seems to be related to individuals' life satisfaction (26). Patients in a depressive mood state are more likely to be dissatisfied with their life, and they seems more easily to have suicidal ideations which linked to poor health related outcomes (17, 27). Notably, the researchers pointed out that biomarkers were correlated with a decline in the functioning of patients with BD (28). Levels of albumin, triglyceride and apolipoprotein have been pointed to predict psychosocial functioning but there are still insufficient investigations (29). Much more studies are necessary.

Thus, the study aimed to: (i) identify discrete functional subgroups in patients with BD based on the FAST domain scores; (ii) compare different subgroups with respect to sociodemographic, clinical, biochemical and neurocognitive variables; (iii) explore potential factors affecting the psychosocial functioning. We hypothesized that: (i) there may be three functional subgroups in fully or partially euthymic patients with BD; (ii) these different functional subgroups will perform differently on some sociodemographic, clinical, biochemical and neurocognitive variables; (iii) the age of first onset, suicidal ideation, degree of depression, number of episodes and biomarker levels may correlate with functional outcomes.

## Materials and methods

### Participants

A total of 140 fully or partially remitted patients with BD aged from 16 to 60 years participated in the cross-sectional study between April 2019 and October 2020 from the wards of the psychiatric department and outpatient department of Xiamen Xianyue Hospital, the Affiliated Brain Hospital of Guangzhou Medical University and the Third Affiliated Hospital of Sun Yat-Sen University, Guangzhou and Xiamen, China. And the diagnosis of BD was done by one psychiatrist who did not involve in the neuropsychological assessments. All patients met the following inclusion criteria: (i) confirmed diagnosis of BD according to DSM-IV (Diagnostic and statistical manual of mental disorders—IV); (ii) be in fully or partially remitted state for at least 3 months, which was defined as a score ≤ 14 on the Hamilton Depression Rating Scale-17 (*HAMD*) (30) and of ≤ 14 on the Young Mania Rating Scale (*YMRS*) (31); (iii) satisfy the basic communicated skills, with voluntary participation in the study. The exclusion criteria were: (i) intelligence quotient (*IQ*) ≤ 70 on Wechsler Adult Intelligence Scale (*WAIS-CR*); (ii) with any other comorbidities or psychiatric disease affecting neuropsychological performance; (iii) accompanied by other serious physical diseases, such as severe organ failures, malignant tumors, etc.; (iv) history of head trauma; (v) substance use disorders; (vi) color blindness or weakness, or with hearing problems; (vii) received electroconvulsive therapy (*ECT*) over the past year; (viii) pregnancy or breastfeeding. All patients maintained previous pharmacological treatment because of the ethical principles. In addition, 70 healthy individuals were recruited from two communities in Guangzhou city as the healthy controls (*HCs*) without any psychiatric disease and there was no family psychiatric history. All participants gave written informed consent prior to their participation in the study. This study obtained approval from the research ethics committee of the Sun Yat-sen University.

### Assessments

#### Sociodemographic and clinical assessment

All the following data were collected by a clinical interview based on the Structured Clinical Interview for DSM-V (*SCID*). The sociodemographic data included age, gender, only-child status, marital status, residence, employment and education level. Clinical data include BMI (*kg/m*^2^), psychotic symptoms, family psychiatric history, past psychiatric history, age at first onset, age of first treatment, chronicity, the number of hospitalizations and medications. Current psychotic symptoms were measured by one psychiatrist who did not involve in the neuropsychological assessments. The YMRS and HAMD scales were used to determine the degree of mania and depression by clinical specialists in psychiatry.

#### Psychosocial functioning assessment

The FAST was used to measure psychosocial functioning in the study, which is an interviewer-administered instrument developed to assess the main difficulties in daily life (32). The FAST contains six specific domains of functional areas, including autonomy, occupational functioning, cognitive functioning, financial issues, interpersonal relationships and leisure time. There are 24 items, and each item is rated using a four-point scale, 0 indicates no difficulty, 1 indicates mild difficulty, 2 indicates moderate difficulty and 3 indicates severe difficulty. The total score is ranging from 0 to 72, and the higher the score, the poorer the functional outcomes. The FAST scale has been widely used in Chinese patients with BD with good reliability and validity (16).

#### Neurocognitive functioning assessment

The neuropsychological battery tests were used to evaluate four different cognitive domains: (i): Attention and processing speed was evaluated with the Digit Symbol Coding and Digital Span Forward subtest and the Trail Making Test Part A (*TMT-A*) (33). (ii): Working memory consisted of the Digital Span Backward subtest of the WAIS-RC and Trail Making Test Part B (*TMT-B*) (34); (iii): Visual memory was tested by the Visual Reproduction subtest and the Visual Recognition subtest of the Wechsler Memory Scale-Revised (*WMS-R*) (35); (iv): Executive function was evaluated by the Stroop Color and Word Test (*SCWT*) (36).

#### Suicidal ideation

The suicidal ideation was evaluated by the Beck scale for suicide ideation scale (*BSI*) (37), which contains 19 items and includes two dimensions: suicidal ideation and suicidal behavior, with adopting 3-point Likert scale ranging from 0 to 2. The first five items were used to test the suicidal ideation in the present study, and the patients answered only the first five questions. If the answers to item 4 and item 5 were “No”, it was considered that there was no suicidal ideation. The BSI scale-Chinese version was demonstrated to be a reliable instrument for individuals with BD (38).

#### Biochemical index assessment

In the study, a total of 9 biomarkers were collected based on previous investigations (18, 29, 39), including total protein, TP; albumin, ALB; lipid metabolism (triglycerides, TG; low-density lipoprotein cholesterol, LDL; high-density lipoprotein cholesterol, HDL); serum thyroxine (free triiodothyronine, FT3; total triiodothyronine, TT3; free thyroxine, FT4; serum total thyroxine, TT4). All individuals were taken fasting blood samples about 5ml at 7:00 am and then placed in a blood collection tube with special measuring instruments by professionals from three hospitals. Specific analyses, such as blood centrifugation and serum extraction of these biomarkers, were performed by relevant laboratories.

### Statistical analysis

The IBM SPSS Statistics 21.0 was used for data analysis. The means and standard deviations (*SD*) were used to describe continuous variables, or the number (*N*) and percentage (%) were used for categorical variables. To compare the baseline data between patients with BD and HCs, a *t*-test was evaluated for continuous variables and the χ^2^ test was used for categorical variables. The neurocognitive tests scores and the FAST scores were switched into the standard values *via z*-scores based on the healthy controls' performance by using the following formula: z-score = (*patients test score—HC test M*)/*HC test SD*. Higher scores of the FAST domains meant lower functioning. On the contrary, the z-scores were reversed, and higher scores indicated better functional performance. Likewise, higher scores of the TMT-A and TMT-B implied worse cognitive performances, which were also inversed that lower scores indicated poorer performance aligned with the other neurocognitive measures.

Then, hierarchical cluster analysis was used to identify functional subgroups in remitted patients with BD. Ward's linkage was chosen as the agglomeration procedure, in addition, counting similarities between samples by squared Euclidean distance. The inspection of the dendrogram (Supplementary material 1) was used to determine the number of clusters and then a discriminant functional analysis (*DFA*) was adopted to validate the clusters. One-way analysis of variance (*ANOVA*) was carried out to analyze differences on six FAST domains among the three resulting groups (lower functioning, LF; moderate functioning, MF; good functioning, GF) and the HC group. When the ANOVA showed significant differences then LSD *post hoc* comparisons were used to examine pairwise differences. The Kruskal Wallis test and ANOVA were applied as appropriate to determine differences in sociodemographic, clinical, neurocognitive and biochemical variables among the three subgroups. Ultimately, the associations between FAST domains and sociodemographic, neurocognitive and biochemical variables were evaluated by Pearson partial correlations controlling for the HAMD and age as covariates. The Pearson correlations were used to determine the associations between FAST domains and clinical variables. All *P*-values were two-tailed, and statistical significance was set to *P* < 0.05.

## Results

### Characteristics of patients with bipolar disorder and healthy controls

Table 1 displays the sociodemographic and clinical characteristics of participants in the study. There were no significant differences between patients with BD (*n* = 140) and HCs (*n* = 70) groups on age, BMI and gender. Moreover, there were significantly different between the two groups on some variables, see details in Table 1. Moreover, antipsychotics [117(83.6%)], lithium [55 (39.3%)], valproate [77 (55.0%)] and benzodiazepines [40 (28.6%)] were commonly used medications in patients with BD. As we expected, the total FAST score of patients with BD (13.35 ± 11.69) was significantly higher than those in the HC group (*P* < 0.001), which indicating a better psychosocial functioning for HCs. In addition, other domains of the FAST in the HC group, except for autonomy and leisure time, performed better than those in the BD group.

**Table 1:** Descriptive summary between patients with bipolar disorder and healthy controls.

			**Statistical analyses**	
	**BD (*N* = 140)**	**HCs (*N* = 70)**	***t***	***p***
	***Mean* (*SD*)**	***Mean* (*SD*)**		
Age (*years*)	31.48 (12.91)	31.51 (9.39)	−0.023	0.982
BMI *(kg/m^2^*)	22.76 (5.04)	22.70 (4.51)	0.087	0.931
Age of first onset (*years*)	23.14 (9.39)	-	-	-
Age of first treatment (*years*)	24.86 (9.63)	-	-	-
Chronicity (*years*)	8.89 (8.75)	-	-	-
Hospitalizations	5.43 (9.08)	-	-	-
HAMD score	6.18 (4.42)	2.00 (2.30)	9.015	<0.001**
YMRS score	3.96 (3.24)	1.04 (2.48)	7.232	<0.001**
**Functioning assessment short test**
*Total score*	13.35 (11.69)	7.69 (8.64)	3.962	<0.001**
*Autonomy*	1.85 (2.03)	1.41 (1.70)	1.640	0.103
*Occupational*	3.64 (3.75)	1.54 (2.81)	4.544	<0.001**
*Cognitive*	2.53 (2.79)	1.61 (2.27)	2.377	0.018*
*Financial*	1.09 (1.52)	0.59 (1.17)	2.629	0.009**
*Interpersonal*	3.30 (3.74)	1.83 (2.69)	3.268	0.001**
*Leisure time*	0.94 (1.32)	0.70 (1.05)	1.441	0.151
	*N* (%)	*N* (%)	χ^2^	*P*
Gender (*female*)	68 (48.6%)	43 (61.4%)	0.686	0.408
Only-child (*yes*)	26 (18.6%)	28 (40.0%)	49.543	<0.001**
Employment (*no*)	113 (80.7%)	14 (20.0%)	9.219	0.002**
**Education**			93.171	<0.001**
≤ Primary school	9 (6.4%)	2 (2.9%)		
Middle school	102 (72.9%)	23 (32.9%)		
≥College	29 (20.7%)	45 (64.2%)		
**Marital status**			18.305	<0.001**
Single	94 (67.1%)	42 (60.0%)		
Non-single	46 (32.9%)	28 (40.0%)		
**Residence**			56.314	<0.001**
Alone	6 (4.3%)	13 (17.1%)		
Live with families	59 (42.1%)	41 (58.6%)		
Live with others	75 (53.6%)	16 (22.9%)		
Psychotic symptoms (*yes*)^a^	34 (24.3%)	-	-	-
Suicidal ideation (*yes*)	29 (20.7%)	-	-	-
Family psychotic history (*yes*)	24 (17.1%)	-	-	-
Past history *(yes*)	18 (12.9%)	-	-	-
**Medicine**			-	-
*Lithium*	55 (39.3%)	-	-	-
*Valproate*	77 (55.0%)	-	-	-
*Lamotrigine*	9 (6.4%)	-	-	-
*Oxcarbazepine*	18 (12.9%)	-	-	-
*Antipsychotics*	117 (83.6%)	-	-	-
*Antidepressants*	7 (5.0%)	-	-	-
*Benzodiazepines*	40 (28.6%)	-	-	-
*Benzhexol*	8 (5.7%)	-	-	-
*Propranolol*	7 (5.0%)	-	-	-
*Others^*b*^*	17 (12.1%)	-	-	-

**p < 0.01; *p < 0.05.

YMRS, Young Mania Rating Scale; HDRS, Hamilton Depression Scale; FAST, Functioning Assessment Short Test; BD, Bipolar Disorder; HCs, Healthy Controls; GF, good functioning; MF, moderate functioning; LF, lower functioning; Hospitalizations means the number of hospitalizations. Data are given as mean (standard deviation); Psychotic symptoms (yes)^a^: 24.3% missing. Others^b^: Hydrotalcite; Benzbromarone; Compound Glycyrrhizin Tablets; Acarbose; Omeprazole; Simvastatin; Atorvastatin; Teprenone; Polyene Phosphatidylcholine; Coenzyme Q10 Capsules.

### Clustering in the FAST in patients with bipolar disorder

**Figure 1:**
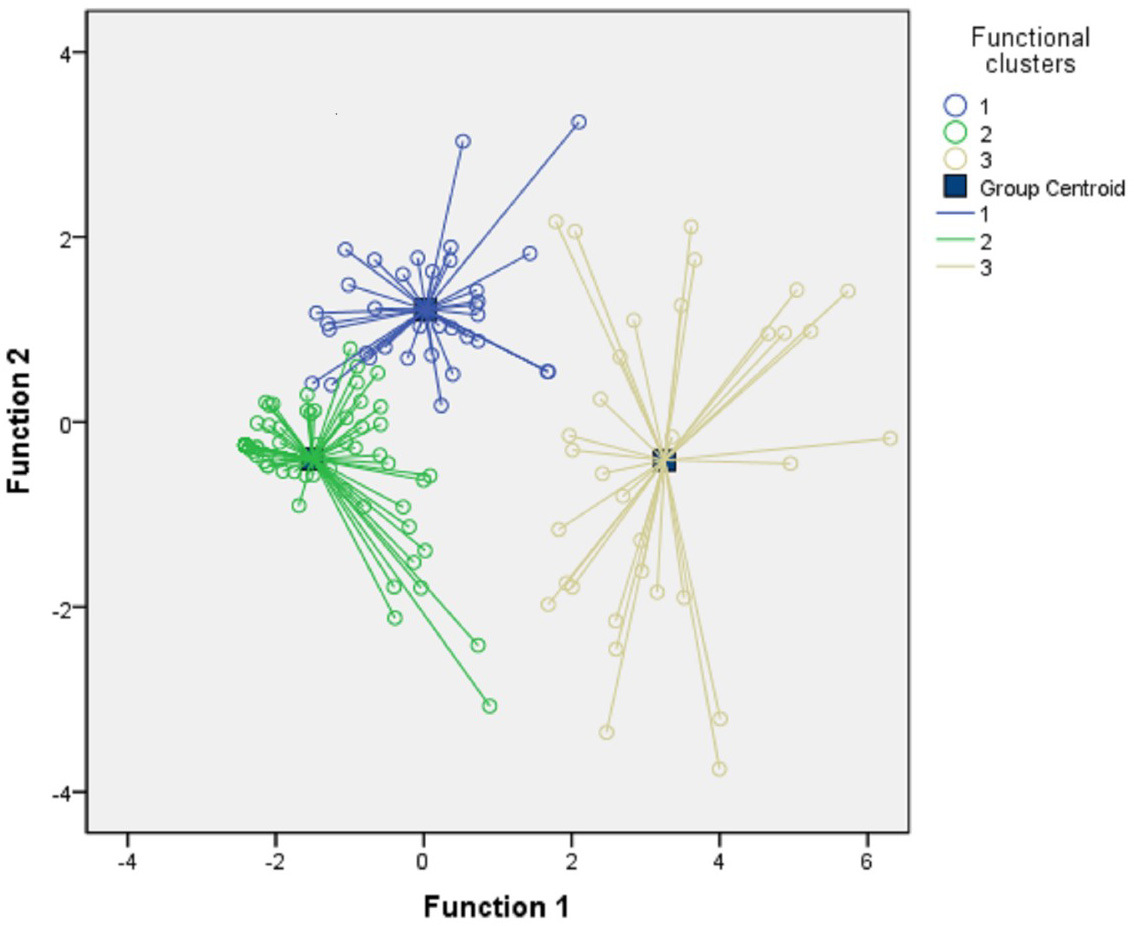
Graphical agglomeration of functional subgroups in patients with BD. Scatter-plot and centroids of the discriminant values vie the discriminant functional analysis (DFA) based on the functional subgroups established from the hierarchical cluster analysis. The centroids are the mean score of three different clusters. Cluster 1 is the moderate functioning group (1 = MF), cluster 2 is the good functioning subgroup (2 = GF) and cluster 3 is the lower functioning group (3 = LF).

Based on the hierarchical cluster analysis, Figure 1. shows that there existed three significantly different functional subgroups in patients with BD. And the three clusters contained 35 patients (25%), 72 patients (51.4%) and 33 patients (23.6%), respectively. The DFA was introduced to check the validity of the three clusters, which revealed the presence of two discriminant functions explaining 88.1% and 11.9% of the variance (Wilks' λ = 0.140, χ^2^ = 264.164, *P* < 0.001; Wilks' λ = 0.665, χ^2^ = 54.924, *P* < 0.001, respectively). Importantly, both financial and occupational domains contributed more than other FAST domains to classify these individuals into different functional clusters. The correlation coefficients for function 1: financial = 0.574, occupational = 0.525; and for function 2: financial = −0.687, occupational = 0.553. According to the DFA, approximately 94.3% of the original samples were grouped into correct functional clusters, which suggests the good validity of hierarchical clusters.

### Comparisons on the FAST domains between three functional clusters and HCs

**Figure 2:**
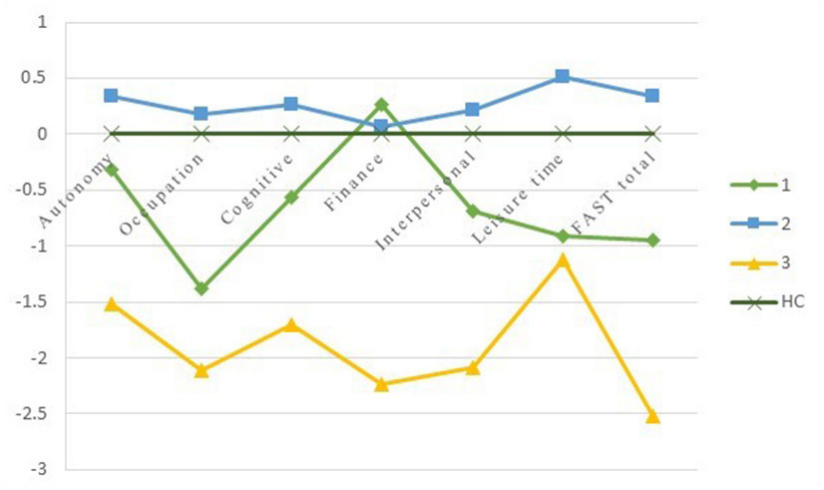
The Z-scores of the FAST comparing BD clusters with HCs. The Y-axis depicts z-scores with a Mean of 0 and SD of 1. HC = healthy controls; cluster 1 = moderate functioning; cluster 2 = good functioning; cluster 3 = lower functioning.

One-way ANOVA displayed significant differences between three clusters and HCs with all *P* < 0.001. Based on the level of functional impairment, the following showed how BD clusters are labeled (Table 2; Figure 2): Cluster 1, the moderate functioning group (*MF*), showed selective impairment compared with HCs for cognitive, occupational, interpersonal and leisure time domains (all *P* < 0.05). Otherwise, all FAST domain scores were higher than those of the LF group with all *P* < 0.001 except for the leisure time, and lower than those of the GF group except for the financial domain. Cluster 2, the good functioning group (*GF*), which performed comparably to HCs in all domains, except for both autonomy and leisure time (*P* = 0.036, *P* = 0.003, respectively). The GF group displayed significantly superior performance than the LF and MF groups in all domains with all *P* < 0.05, except for the financial domain between the GF and MF groups. Cluster 3, the lower functioning group (*LF*), performed a global functioning impairment in all FAST domains, with significantly inferior performance in comparison with HCs and GF groups with all *P* < 0.001, and presented inferior performance than the MF group on most FAST domains (all *P* < 0.05), except for the leisure time.

**Table 2:** Comparisons between FAST domains according to three functional clusters and HCs (*Z*-score).

	**Clusters**						
	**LF (*n* = 33)**	**MF (*n* = 35)**	**GF (*n* = 72)**	**HC (*n* = 70)**	***F***	***P***	***Post hoc tests***
Autonomy	−1.51 (1.24)	−0.32 (0.95)	0.34 (0.77)	0.00 (1.00)	29.19	<0.001**	**HC** **ν. GF** ***P** **=*** 0.036*
							HC ν. MF *P =* 0.115
							**HC** **ν. LF** ***P** **<*** 0.001**
							**GF** **ν. MF** ***P** **=*** 0.001**
							**GF** **ν. LF** ***P** **<*** 0.001**
							**MF** **ν. LF** ***P** **<*** 0.001**
Occupational	−2.11 (1.22)	−1.38 (1.16)	0.17 (0.56)	0.00 (1.00)	60.76	<0.001**	HC ν. MF *P =* 0.275
							**HC** **ν. MF** ***P** **<*** 0.001**
							**HC** **ν. LF** ***P** **<*** 0.001**
							**GF** **ν. MF** ***P** **<*** 0.001**
							**GF** **ν. LF** ***P** **<*** 0.001**
							**MF** **ν. LF** ***P** **=*** 0.002**
Cognitive	−1.71 (1.39)	−0.57 (1.00)	0.26 (0.63)	0.00 (1.00)	34.51	<0.001**	HC ν. GF *P =* 0.105
							**HC** **ν. MF** ***P** **=*** 0.005**
							**HC** **ν. LF** ***P** **<*** 0.001**
							**GF** **ν. MF** ***P** **<*** 0.001**
							**GF** **ν. LF** ***P** **<*** 0.001**
							**MF** **ν. LF** ***P** **<*** 0.001**
Financial	−2.23 (1.23)	0.26 (0.49)	0.07 (0.75)	0.00 (1.00)	62.36	<0.001**	HC ν. GF *P =* 0.663
							HC ν. MF *P =* 0.159
							**HC** **ν. LF** ***P** **<*** 0.001**
							GF ν. MF *P =* 0.288
							**GF** **ν. LF** ***P** **<*** 0.001**
							**MF** **ν. LF** ***P** **<*** 0.001**
Interpersonal	−2.08 (1.46)	−0.68 (1.25)	0.22 (0.65)	0.00 (1.00)	42.00	<0.001**	HC ν. GF *P =* 0.212
							**HC** **ν. MF** ***P** **=*** 0.002**
							**HC** **ν. LF** ***P** **<*** 0.001**
							**GF** **ν. MF** ***P** **<*** 0.001**
							**GF** **ν. LF** ***P** **<*** 0.001**
							**MF** **ν. LF** ***P** **<*** 0.001**
Leisure time	−1.12 (1.46)	−0.91 (1.31)	0.51 (0.42)	0.00 (1.00)	27.80	<0.001**	**HC** **ν. GF** ***P** **=*** 0.003**
							**HC** **ν. MF** ***P** **<*** 0.001**
							**HC** **ν. LF** ***P** **<*** 0.001**
							**GF** **ν. MF** ***P** **<*** 0.001**
							**GF** **ν. LF** ***P** **<*** 0.001**
							MF ν. LF *P =* 0.388
FAST total	−2.52 (1.00)	−0.95 (0.75)	0.33 (0.52)	0.00 (1.00)	102.73	<0.001**	**HC** **ν. GF** ***P** **=*** 0.017*
							**HC** **ν. MF** ***P** **<*** 0.001**
							**HC** **ν. LF** ***P** **<*** 0.001**
							**GF** **ν. MF** ***P** **<*** 0.001**
							**GF** **ν. LF** ***P** **<*** 0.001**
							**MF** **ν. LF** ***P** **<*** 0.001**

**p < 0.01; *p < 0.05.

HC, healthy controls; GF, good functioning; MF, moderate functioning; LF, lower functioning. Data are given as mean (standard deviation).

### Comparisons on sociodemographic, clinical, neurocognitive and biochemical variables among the three functional subgroups

As shown in Table 3, ANOVA displayed significant differences among the three groups on age (*P* = 0.046), then the pairwise comparisons revealed that patients in the GF group were significantly older than patients in the LF group (*P* = 0.023). Regarding clinical variables, three subgroups did differ on the HAMD scores (*P* = 0.011), with the LF group presenting more depressive symptoms than the GF group (*P* = 0.004). There were significant differences among the three groups on the age of first onset (*P* = 0.043), where the age of first onset of patients in the LF group appeared to be younger than those of patients in the GF group (*P* = 0.016). Notably, the patients performed differently in terms of suicidal ideation regarding *P* = 0.045, with *post hoc* tests revealing that the prevalence of patients who had suicidal ideation in the MF group was higher than those in the GF group (*P* = 0.023). Concerning neurocognitive variables, there were no differences on most of neurocognitive domains, except for the visual reproduction test between the GF and MF groups (*P* = 0.042) (see Table 4).

**Table 3:** Comparisons in sociodemographic, clinical and biochemical variables between the three functional clusters.

	**Cluster**							
	**GF (*n* = 72)**	**MF (*n* = 35)**	**LF (*n* = 33)**	***F*/*H***	***P***	***Post hoc tests***		
	***M* (*SD*)/*N* (%)**	***M* (*SD*)/*N* (%)**	***M* (*SD*)/*N* (%)**			**GF ν. MF**	**GF ν. LF**	**MF ν. LF**
**Sociodemographic variables**
Age (*years*)	34.03 (12.82)	29.63 (13.78)	27.88 (11.22)	3.139	0.046*	0.095	0.023*	0.572
BMI (*kg/m^2^*)	22.89 (4.73)	22.62 (4.47)	22.63 (6.29)	0.049	0.952			
Sex				3.918	0.141			
Male	40 (55.6%)	20 (57.1%)	12 (36.4%)					
Female	32 (44.4%)	15 (42.9%)	21 (63.6%)					
Only-child (*yes*)	15 (20.8%)	5 (14.3%)	6 (18.2%)	1.103	0.576			
Marial status				3.225	0.199			
Single	44 (61.1%)	24 (68.6%)	26 (78.8%)					
Non-single	28 (38.9%)	11 (31.4%)	7 (21.2%)					
Residence				0.537	0.765			
Alone	3 (4.2%)	2 (5.7%)	1 (3.0%)					
Live with families	29 (40.3%	16 (45.7%)	14 (42.4%)					
Live with others	40 (55.6%)	17 (48.6%)	18 (54.5%)					
Employment (*no*)	54 (75.0%)	30 (85.7%)	29 (87.9%)	3.138	0.208			
Education				2.656	0.265			
≤ Primary school	4 (5.6%)	3 (8.6%)	2 (6.1%)					
Middle school	50 (69.4%)	28 (80.0%)	24 (72.7%)					
≥College	18 (25.0%)	4 (11.4%)	7 (21.2%)					
HAMD score	5.18 (4.00)	6.66 (3.53)	7.85 (5.53)	4.672	0.011*	0.099	0.004**	0.256
YMRS score	3.74 (3.11)	3.71 (2.89)	4.73 (3.83)	1.198	0.305			
**Clinical variables**
Psychotic symptoms (*yes*)	15 (20.8%)	9 (25.7%)	10 (30.3%)	0.660	0.719			
Family psychotic history (*yes*)	14 (19.4%)	6 (17.1%)	4 (12.1%)	0.848	0.654			
Past history (*yes*)	10 (13.9%)	5 (14.3%)	3 (9.1%)	0.546	0.761			
Age of first onset (*years*)	24.93 (9.14)	22.23 (1.49)	20.18 (7.99)	3.209	0.043*	0.158	0.016*	0.363
Age of first treatment (*years*)	26.47 (9.52)	24.23 (10.24)	22.03 (8.73)	2.563	0.081			
Chronicity (*years*)	9.71 (9.87)	7.94 (7.43)	8.12 (7.42)	0.644	0.527			
Hospitalizations	5.43 (9.19)	6.17 (9.58)	4.64 (9.20)	0.232	0.794			
Number of manic episodes	5.96 (9.35)	12.17 (24.73)	10.94 (23.34)	1.110	0.334			
Number of depressed episodes	0.95 (1.38)	7.33 (19.60)	7.07 (23.32)	1.877	0.160			
Number of mixed episodes	0.23 (0.43)	5.79 (20.10)	7.46 (24.23)	1.675	0.195			
Suicidal ideation (*yes*)	9 (12.5%)	11 (31.4%)	9 (27.3%)	6.224	0.045*	0.023*	0.081	0.669
**Biochemical index**
*FT3 (pmol/ml)*	4.64 (0.88)	4.50 (0.58)	4.61 (0.58)	0.314	0.732			
*TT3 (nmol/L)*	1.99 (1.74)	1.36 (0.19)	1.55 (0.24)	1.440	0.250			
*FT4 (pmol/ml)*	3.04 (11.26)	14.09 (11.51)	11.26 (3.04)	1.494	0.230			
*TT4 (nmol/L)*	90.28 (25.17)	85.43 (29.47)	108.75 (14.41)	3.018	0.062	0.614	0.081	0.023*
*TP (g/L)*	65.79 (12.01)	67.80 (11.73)	68.24 (5.14)	0.769	0.465			
*ALB (g/L)*	40.41 (7.93)	43.69 (3.86)	42.45 (3.99)	3.160	0.046*	0.016*	0.157	0.433
*TG (mmol/L)*	1.61 (0.75)	1.33 (0.90)	1.20 (0.55)	3.097	0.049*	0.097	0.023*	0.512
*HDL (mmol/L)*	1.16 (0.28)	1.12 (0.23)	1.29 (0.34)	2.889	0.060	0.506	0.054	0.023*
*LDL (mmol/L)*	2.71 (0.68)	2.65 (0.79)	2.74 (0.55)	0.135	0.874			

**p < 0.01; *p < 0.05.

YMRS, Young Mania Rating Scale; HAMD, Hamilton Depression Scale; FAST, Functioning Assessment Short Test; BD, Bipolar Disorder; HCs, Healthy Controls; GF, good functioning; MF, moderate functioning; LF, lower functioning; FT3, Free triiodothyronine; TT3, Total triiodothyronine; FT4, Free thyroxine; TT4, Serum total thyroxine; TP, Total protein; ALB, Albumin; TG, Triglyceride; HDL, High-density lipoprotein cholesterol; LDL, Low-density lipoprotein cholesterol.

**Table 4:** Comparisons between three functional clusters on neurocognitive variables (z-score).

	**GF** **(*n* = 72)** ***Mean (SD)***	**MF** **(*n* = 35)** ***Mean (SD)***	**LF** **(*n* = 33)** ***Mean (SD)***			***Post hoc test***		
				***F***	***P***	**GF ν. MF**	**GF ν. LF**	**MF ν. LF**
**Attention and Processing speed**	−0.96 (1.15)	−0.83 (1.31)	−0.74 (1.21)	0.392	0.676			
Digital span forward subtest	−0.22 (1.30)	−0.03 (0.83)	−0.20 (1.40)	0.303	0.739			
TMT-A	1.46 (2.16)	1.39 (2.76)	1.16 (2.10)	0.194	0.824			
Digit symbol coding subtest	−1.19 (1.08)	−1.07 (1.14)	−0.87 (1.28)	0.927	0.398			
**Working memory**	−1.04 (1.30)	−0.95 (1.26)	−1.21 (1.55)	0.322	0.725			
TMT-B	1.75 (2.10)	1.57 (1.28)	1.87 (2.55)	0.149	0.862			
Digital span backward subtest	−0.33 (0.90)	−0.32 (0.77)	−0.55 (0.84)	0.823	0.441			
**Visual memory**	−0.81 (1.29)	−0.37 (1.26)	−0.52 (1.47)	1.437	0.241			
Visual recognition subtest	−0.49 (1.47)	−0.32 (1.36)	−0.18 (1.63)	0.540	0.584			
Visual reproduction subtest	−1.12 (1.59)	−0.42 (1.64)	−0.86 (1.85)	2.102	0.126	0.042*	0.466	0.270
**Executive function**	−0.91 (1.02)	−0.76 (0.97)	−0.65 (0.98)	0.856	0.427			
Stroop A	−1.36 (1.51)	−1.05 (1.30)	−0.92 (1.52)	1.242	0.292			
Stroop B	−0.74 (1.06)	−0.67 (1.07)	−0.52 (0.98)	0.511	0.601			
Stroop C	−0.63 (0.89)	−0.58 (0.86)	−0.50 (0.98)	0.231	0.794			

*p < 0.05.

GF, good functioning; MF, moderate functioning; LF, lower functioning; TMT-A, Trail Making Test-part A; TMT-B, Trail Making Test-part B; Stroop A/B/C, Stroop Color-Word Test.

Specifically, concerning biochemical variables, there were statistically significant differences among the three groups in ALB and TG levels (*P* = 0.046, *P* = 0.049). The pairwise comparisons indicated that the ALB level in the MF group was higher than those in the GF group (*P* = 0.016) and the TG level in the LF group was lower than those in the GF group (*P* = 0.023). In addition, compared with the MF group, the levels of TT4 and HDL in the LF group were significantly higher (all *P* = 0.023). Otherwise, there showed no differences on other variables among the three subgroups with all *P* values >0.05.

### Associations between FAST domains and sociodemographic, clinical, neurocognitive and biochemical variables within each functional subgroup

The HAMD scores and age were controlled based on previous studies which confirmed depressive symptoms could affect psychosocial functioning (15, 40). In the GF group, the TMT-A was positively correlated with the cognitive domain (*r* = 0.703, *P* = 0.035). Furthermore, the FT4 and Stroop A were correlated with the financial domain (*r* = −0.693, *P* = 0.039; *r* = 0.750, *P* = 0.020). Concerning the MF group, the TMT-B and working memory were positively correlated with the leisure time domain (*r* = 0.623, *P* = 0.023; *r* = 0.587, *P* = 0.035). The visual recognition test and visual memory were positively correlated with the autonomy (*r* = 0.556, *P* = 0.048; *r* = 0.576, *P* = 0.040). The interpersonal domain was positively correlated with the Digital span forward subtest (*r* = 0.600, *P* = 0.030) and negatively correlated with ALB and TP (*r* = −0.773, *P* = 0.002; *r* = −0.640, *P* = 0.018). The TT4 and FT4 were correlated with the financial domain (*r* = 0.809, *P* = 0.001; *r* = −0.616, *P* = 0.025). Finally, in the LF group, the working memory was positively correlated with the autonomy (*r* = 0.794, *P* = 0.011). The Visual reproduction test, TT3 and TT4 were correlated with the leisure time domain (*r* = −0.736, *P* = 0.024; *r* = 0.736, *P* = 0.024; *r* = 0.669, *P* = 0.049). Moreover, the Stroop B, executive function and LDL were positively correlated with the occupational domain (*r* ranging from 0.745 to 0.806, *P* from 0.009 to 0.028).

The associations between clinical variables and FAST domains. Concerning the GF group, the age of first onset was negatively associated with the financial domain (r ranging from −0.624 to −0.321). With regard to the MF group, hospitalizations were positively correlated with both autonomy and leisure time domains (*r* = 0.455, *P* = 0.006); the age of first treatment negatively correlated with both occupational and leisure time domains (*r* = −0.359, *P* = 0.034; *r* = −0.342, *P* = 0.044, respectively); the number of mixed episodes were positively associated with the cognitive domain (*r* = 0.516, *P* = 0.024); the age of first treatment was positively correlated with the leisure time (*r* = 0.396, *P* = 0.019). In the LF group, the number of manic/depressed/mixed episodes was moderately correlated with the interpersonal domain (r ranging from 0.603 to 0.668). The correlations were not found in any other rest clinical variables in the three subgroups.

## Discussion

In the present study, we confirmed that there were three functional subgroups, ranging from the good functioning group, moderate functioning group to lower functioning group (GF, MF and LF), according to their functional performances compared with HCs, which were aligned with previous studies (15, 41). Additionally, approximately 48.6% of patients with BD performed functional impairments which was consistent with previous studies (42–44). As expected, several variables presented differently among the three subgroups (LF, MF and GF), such as the employment status, age, HAMD scores, age of first onset, suicidal ideation, several neurocognitive variables and biochemical indexes (TT3, LDL, FT4, TT4, TP and ALB) in patients with BD.

Notably, around 80.7% of patients with BD were absent from work in the present study, which was significantly different from HCs, only 20.0% of whom were unemployed. The higher unemployment rate in patients with BD might be influenced by lower psychosocial functioning that would affect the patients' working capacities and interpersonal communications, increasing the risks of hiring and quitting jobs. Patients with BD can barely control situations that worsen their life satisfaction (6, 45), thus, helping patients to obtain a better working experience might alleviate the aggravation of functional impairments.

The financial and occupational domain of the FAST contributed more to classify functional subgroups. Although the contribution of the financial domain did not align with previous research, the role of the occupational domain was entirely consistent with an earlier study (15). Importantly, we found that executive function was positively correlated with the occupational domain within the LF group, revealing that the executive functional impairment affects the patients' occupational function. Executive function is a commonly and mainly influenced domain of cognitive function which is significantly related to our daily life (46, 47). Furthermore, the executive function could significantly predict the employment status in patients with BD (48). Therefore, occupational dysfunction should be emphasized in functional outcomes of patients with BD (49). The results also revealed that the number of episodes moderately influenced the cognitive and interpersonal function in the LF and MF groups, which were also similar to Sole et al. (15).

Based on our results, three functional subgroups (LF, MF and GF) did differ on age. Patients with BD in the LF group were younger than those in the GF group, indicating the younger patients with BD present poor psychosocial functioning, inconsistent with previous studies (40, 50). The possible explanation is that a relatively large proportion of juveniles ranging from 16–17 years old in the LF group (15.2%) in our study, most of them are unable to adapt to society well, especially present a poor occupational ability. The other explanation is that more depressive symptoms are positively correlated with poor psychosocial functioning in patients with bipolar disorder (27), and the HAMD scores of the LF group were higher than the MF group in our study, which might bring out a poorer functioning. In addition, the age of first onset of patients in the LF group is lower than individuals in the GF group, which has been confirmed negatively correlate with poor functional outcomes (20, 51). Another important point, we found around 20.7% patients with BD reported suicidal ideation in our study, consistent with other clinical research (52). Besides, the suicidal ideation of the MF group performed stronger than that of the GF group, 31.4 and 12.5% respectively, which implied that patients with suicidal ideations seems more easily to arise functioning deficits. We thought it may be related to life satisfaction, because functional impairment hinders patients from returning to normal social life, just like leading to worse interpersonal relationships and worse occupational situations, all of which have already been proven by others (53, 54).

For neurocognitive functioning, Unexpectedly, no significant differences were found in neurocognitive functions among different functional profiles in our study. Maybe it is because the sample of patients in our study was relative young (31.48 ± 12.91 years old), small (*n* = 140) and short chronicity (8.89 ± 8.75 years), which might affect the inconspicuous neurocognitive decline in patients with bipolar disorder (55, 56). Therefore, a relative big sample and large age span sample of patients with bipolar disorder are needed in further studies. Results from Pearson partial correlation showed that executive function, working and visual memory did moderately correlated with functional domains which is consistent with one previous research (14). However, there still exists controversies about which neurocognitive domains could explain the heterogeneity of functional outcomes (57).

Preliminary as it was, we still found some significant results regarding biochemical indexes. The levels of ALB, TG, TT4 and HDL performed differently among the three groups. Notably, the TG levels of patients with BD in the GF group were higher than the normal range (1.70–2.26 mmol/L), while the ALB levels of patients with BD in the GF group were lower than the normal range (35–51 g/L). The drug-protein interactions revealed olanzapine (OLZ) will reduce ALB abundance for increasing medication effect (58). Hence, the extensive use of olanzapine may be one of the possible reasons for the low ALB levels in the GF group. Another possible explanation for high TG levels and lower ALB levels is medical comorbidities, especially hypertriglyceridemia and hypoalbuminemia, which might cause functional impairment (18). Individuals of the GF were older than others (see Table 3), and more likely to suffer from comorbidities such as hyperlipidemia. In the study, we found that the TT4 levels of the LF group were higher than those of the MF group, which may indicate poor functioning. According to previous studies, thyroid function played a crucial role in the pathogenesis of bipolar disorder, which may refer to hypothalamic-pituitary-thyroid (HPT) (39, 59). As neuroendocrine indices of HPT, the abnormal changes of T3, T4, FT3, FT4 and other related thyroid hormones were often associated with more episodes and severe depressive symptoms (59, 60), both of them have an influence on psychosocial functioning (3, 27). Interestingly, FT4 and TT4 were correlated with the financial domain of subjects in the MF and GF groups, which might link to the mood changes of HPT involvement in BD that performed depressive or hypomanic/manic behaviors corresponding to hyperthyroidism and hypothyroidism (61). The ALB and TP levels were negatively correlated with interpersonal domain, which may be related to unhealthy physical conditions, such as comorbidities. The mechanism between biochemical indexes and functional outcome is still not clear and a little bit complex. The influences of sample sizes, medical comorbidities, type or dosage of medications and antipsychotic side effects still should be considered, further study is necessary.

## Limitations and strengths

There are several limitations in the study. Firstly, because it was circumscribed to the cross-sectional design, the causal relationship between functioning, sociodemographic, clinical, neurocognitive and biochemical variables, as well as the stability of functional subgroups cannot be evaluated, which means that more longitudinal researches are needed to verify the stability of these subgroups. In addition, we did not control the pharmacological treatment for ethical considerations, including the types and dosages of medications. Thirdly, the BD subtypes and comorbidity conditions were not measured in our study, which might affect bipolar patients' functioning. Finally, the sample size was relatively small and partially remitted patients with BD were recruited in our study which may influence the reliability of the results. Even though there were some limitations, our study verified different functional clusters in Chinese patients with BD, with different functional clusters that exhibited differences in functional performance.

## Conclusions

To sum up, this cross-sectional study successfully identified three specific functional profiles in fully or partially remitted patients with BD based on the FAST scale. Notably, bipolar patients with different functional profiles presented differently in the patterns of several sociodemographic, clinical, neurocognitive and biochemical variables. Thus, psychosocial rehabilitation programs or functional remediation for patients with BD should fully consider this high variability or heterogeneity of psychosocial functioning, as well as the influencing factors in the future.

## Data availability statement

The raw data supporting the conclusions of this article will be made available by the authors, without undue reservation.

## Ethics statement

This study obtained approval from the research ethics committee of the Sun Yat-sen University. The patients/participants provided their written informed consent to participate in this study. Written informed consent was obtained from the individual(s) for the publication of any potentially identifiable images or data included in this article.

## Author contributions

XL designed the study and wrote the protocol. ZB and XL undertook the statistical analysis and wrote the first draft of the manuscript. XL revised the manuscript. XL, DL, and YZ managed the data collection and clinical evaluations. JL, KZ, MC, YL, and HF did the data collection and statistical analysis. All authors contributed to and have approved the final manuscript.

## Funding

This work was supported by the Natural Science Foundation of Guangzhou City (Grant Number 202201011450) and the National Natural Science Foundation of China (Grant Number 71904213).

## Conflict of interest

The authors declare that the research was conducted in the absence of any commercial or financial relationships that could be construed as a potential conflict of interest.

## Publisher's note

All claims expressed in this article are solely those of the authors and do not necessarily represent those of their affiliated organizations, or those of the publisher, the editors and the reviewers. Any product that may be evaluated in this article, or claim that may be made by its manufacturer, is not guaranteed or endorsed by the publisher.

## References

[1] Carvalho AF Firth J Vieta E. Bipolar disorder. N Engl J Med. (2020) 383:58–66. 10.1056/NEJMra1906193 32609982

[2] Anderson IM Haddad PM Scott J. Bipolar disorder. BMJ. (2012) 345:e8508. 10.1136/bmj.e8508 23271744

[3] MacQueen GM Young LT Robb JC Marriott M Cooke RG Joffe RT. Effect of number of episodes on wellbeing and functioning of patients with bipolar disorder. Acta Psychiatr Scand. (2000) 101:374–81. 10.1034/j.1600-0447.2000.101005374.x 10823297

[4] MacQueen GM Young LT Joffe RT. A review of psychosocial outcome in patients with bipolar disorder. Acta Psychiatr Scand. (2001) 103:163–70. 10.1034/j.1600-0447.2001.00059.x 11240572

[5] Bonnín CDM Reinares M Martínez-Arán A Jiménez E Sánchez-Moreno J Solé B. Improving functioning, quality of life, and well-being in patients with bipolar disorder. Int J Neuropsychopharmacol. (2019) 22:467–77. 10.1093/ijnp/pyz018 31093646 PMC6672628

[6] Jiménez-López E Sánchez-Morla EM López-Villarreal A Aparicio AI Martínez-Vizcaíno V Vieta E. Neurocognition and functional outcome in patients with psychotic, non-psychotic bipolar I disorder, and schizophrenia. A five-year follow-up. Eur Psychiatry. (2019) 56:60–8. 10.1016/j.eurpsy.2018.11.008 30500572

[7] Gabriela L Severino B Ângela M. Functioning in euthymic patients with bipolar disorder: a systematic review and meta-analysis using the functioning assessment short test. Bipolar Disord. (2020) 22:569–81. 10.1111/bdi.12904.2904 32243046

[8] Anaya C Torrent C Caballero FF Vieta E Bonnin Cdel M Ayuso-Mateos JL. Cognitive reserve in bipolar disorder: relation to cognition, psychosocial functioning and quality of life. Acta Psychiatr Scand. (2016) 133:386–98. 10.1111/acps.12535 26719018

[9] Haro JM Reed C Gonzalez-Pinto A Novick D Bertsch J Vieta E. 2-Year course of bipolar disorder type I patients in outpatient care: factors associated with remission and functional recovery. Eur Neuropsychopharmacol. (2011) 21:287–93. 10.1016/j.euroneuro.2010.08.001 20956071

[10] Sanchez-Moreno J Martinez-Aran A Vieta E. Treatment of functional impairment in patients with bipolar disorder. Curr Psychiatry Rep. (2017) 19:3. 10.1007/s11920-017-0752-3 28097635

[11] Kremen WS Seidman LJ Faraone SV Toomey R Tsuang MT. The paradox of normal neuropsychological function in schizophrenia. J Abnorm Psychol. (2000) 109:743–52. 10.1037/0021-843X.109.4.743 11196000

[12] Burdick KE Russo M Frangou S Mahon K Braga RJ Shanahan M. Empirical evidence for discrete neurocognitive subgroups in bipolar disorder: clinical implications. Psychol Med. (2014) 44:3083–96. 10.1017/S0033291714000439 25065409 PMC4797987

[13] Jensen JH Knorr U Vinberg M Kessing LV Miskowiak KW. Discrete neurocognitive subgroups in fully or partially remitted bipolar disorder: associations with functional abilities. J Affect Disord. (2016) 205:378–86. 10.1016/j.jad.2016.08.018 27573491

[14] Lomastro MJ Valerio MP Blasco MB Tagni MF Martino DJ. Predictors of high psychosocial functioning in bipolar disorder. J Nerv Ment Dis. (2020) 208:904–7. 10.1097/NMD.0000000000001224 33105443

[15] Sole B Bonnin CM Jiménez E Torrent C Torres I Varo C. Heterogeneity of functional outcomes in patients with bipolar disorder: a cluster-analytic approach. Acta Psychiatr Scand. (2018) 137:516–27. 10.1111/acps.12871 29508379

[16] Zhang Y Long X Ma X He Q Luo X Bian Y. Psychometric properties of the Chinese version of the Functioning Assessment Short Test (FAST) in bipolar disorder. J Affect Disord. (2018) 238:156–60. 10.1016/j.jad.2018.05.019 29883937

[17] Anyayo L Ashaba S Kaggwa MM Maling S Nakimuli-Mpungu E. Health-related quality of life among patients with bipolar disorder in rural southwestern Uganda: a hospital based cross sectional study. Health Qual Life Outcomes. (2021) 19:84. 10.1186/s12955-021-01729-5 33691720 PMC7945052

[18] Dargél AA Volant S Brietzke E Etain B Olié E Azorin JM. Allostatic load, emotional hyper-reactivity, and functioning in individuals with bipolar disorder. Bipolar Disord. (2020) 22:711–21. 10.1111/bdi.12927 32415900

[19] Vierck E Joyce PR. Influence of personality and neuropsychological ability on social functioning and self-management in bipolar disorder. Psychiatry Res. (2015) 229:715–23. 10.1016/j.psychres.2015.08.015 26282228

[20] Grande I Magalhães PV Chendo I Stertz L Panizutti B Colpo GD. Staging bipolar disorder: clinical, biochemical, and functional correlates. Acta Psychiatr Scand. (2014) 129:437–44. 10.1111/acps.12268 24628576

[21] Lewandowski KE Sperry SH Cohen BM Norris LA Fitzmaurice GM Ongur D. Treatment to Enhance Cognition in Bipolar Disorder (TREC-BD): efficacy of a randomized controlled trial of cognitive remediation versus active control. J Clin Psychiatry. (2017) 78:e1242–e9. 10.4088/JCP.17m11476 29045770

[22] Vieta E Popovic D Rosa AR Solé B Grande I Frey BN. The clinical implications of cognitive impairment and allostatic load in bipolar disorder. Eur Psychiatry. (2013) 28:21–9. 10.1016/j.eurpsy.2011.11.007 22534552

[23] Popolo R Borsella I Meschini L Pianella U Zampaglione G Vinci G. Cognitive theory of mind in bipolar disorder: comparisons with healthy controls and associations with function. Psychiatry Res. (2020) 290:113030. 10.1016/j.psychres.2020.113030 32485485

[24] Lin X Lu D Zhu Y Luo X Huang Z Chen W. The effects of cognitive reserve on predicting and moderating the cognitive and psychosocial functioning of patients with bipolar disorder. J Affect Disord. (2020) 260:222–31. 10.1016/j.jad.2019.09.019 31505400

[25] Studart P Galvão-de Almeida A Bezerra-Filho S Caribé A Reis Afonso N Daltro C. Is history of suicidal behavior related to social support and quality of life in outpatients with bipolar I disorder? Psychiatry Res. (2016) 246:796–802. 10.1016/j.psychres.2016.10.045 28029441

[26] Berlim MT Pargendler J Caldieraro MA Almeida EA Fleck MP Joiner TE. Quality of life in unipolar and bipolar depression: are there significant differences? J Nerv Ment Dis. (2004) 192:792–5. 10.1097/01.nmd.0000144700.97769.06 15505526

[27] Anmella G Gil-Badenes J Pacchiarotti I Verdolini N Aedo A Angst J. Do depressive and manic symptoms differentially impact on functioning in acute depression? Results from a large, cross-sectional study. J Affect Disord. (2020) 261:30–9. 10.1016/j.jad.2019.09.070 31600585

[28] Aydemir Ö Çubukçuoglu Z Erdin S Taş C Onur E Berk M. Oxidative stress markers, cognitive functions, and psychosocial functioning in bipolar disorder: an empirical cross-sectional study. Braz J Psychiatry. (2014) 36:293–7. 10.1590/1516-4446-2013-1299 24770657

[29] Łojko D Owecki M Suwalska A. Impaired Glucose Metabolism in Bipolar Patients: The Role of Psychiatrists in Its Detection and Management. Int J Environ Res Public Health. (2019) 16:1132. 10.3390/ijerph16071132 30934836 PMC6480108

[30] Hamilton M. A rating scale for depression. J Neurol Neurosurg Psychiatry. (1960) 23:56–62. 10.1136/jnnp.23.1.56 14399272 PMC495331

[31] Young RC Biggs JT Ziegler VE Meyer DA. A rating scale for mania: reliability, validity and sensitivity. Br J Psychiatry. (1978) 133:429–35. 10.1192/bjp.133.5.429 728692

[32] Rosa AR Sánchez-Moreno J Martínez-Aran A Salamero M Torrent C Reinares M. Validity and reliability of the Functioning Assessment Short Test (FAST) in bipolar disorder. Clin Pract Epidemiol Ment Health. (2007) 3:5. 10.1186/1745-0179-3-5 17555558 PMC1904447

[33] Lezak MD Howieson DB Loring DW. Neuropsychological Assessment. 4th ed. New York, NY: Oxford University Press (2004).

[34] Lu L Bigler ED. Performance on original and a Chinese version of Trail Making Test Part B: a normative bilingual sample. Appl Neuropsychol. (2000) 7:243–6. 10.1207/S15324826AN0704_6 11296687

[35] Gong YX. Wechsler Memory Scale-Revised in China. Hunan: Hunan Medical School Press (1987).

[36] Golden CJ. Stroop Color and Word Test: A Manual for Clinical and Experimental Uses. Wood Dale, IL: Stoelting Co (1978).

[37] Beck AT. BSI, *Beck Scale for Suicide Ideation: Manual*. New York, NY: Psychological Corporation (1991).

[38] Wang LM Shen YL Liang ZQ Luo ZY Zhang KR. Reliability and validity of BSI-CV in evaluating the depression patients. Chin J Health Psychol. (2012) 20:159–60.

[39] Sun XL editor. Consensus of Identification and Optimize Therapeutic Scheme of Bipolar Affective Disorder and Its Atypical Symptoms. The 12th Western psychiatric Forum, Chengdu, Sichuan, China (2015).

[40] Weisenbach SL Marshall D Weldon AL Ryan KA Vederman AC Kamali M. The double burden of age and disease on cognition and quality of life in bipolar disorder. Int J Geriatr Psychiatry. (2014) 29:952–61. 10.1002/gps.4084 24677268

[41] Van Rheenen TE Lewandowski KE Tan EJ Ospina LH Ongur D Neill E. Characterizing cognitive heterogeneity on the schizophrenia-bipolar disorder spectrum. Psychol Med. (2017) 47:1848–64. 10.1017/S0033291717000307 28241891

[42] Kebede D Alem A Shibire T Deyassa N Negash A Beyero T. Symptomatic and functional outcome of bipolar disorder in Butajira, Ethiopia. J Affect Disord. (2006) 90:239–49. 10.1016/j.jad.2005.11.009 16376431

[43] Martino DJ Igoa A Scápola M Marengo E Samamé C Strejilevich SA. Functional outcome in the middle course of bipolar disorder: a longitudinal study. J Nerv Ment Dis. (2017) 205:203–6. 10.1097/NMD.0000000000000583 28234724

[44] Léda-Rêgo G Bezerra-Filho S Miranda-Scippa Â. Functioning in euthymic patients with bipolar disorder: a systematic review and meta-analysis using the Functioning Assessment Short Test. Bipolar Disord. (2020) 22:569–81. 10.1111/bdi.12904 32243046

[45] Henry C Etain B Godin O Dargel AA Azorin JM Gard S. Bipolar patients referred to specialized services of care: Not resistant but impaired by sub-syndromal symptoms. Results from the FACE-BD cohort. Aust N Z J Psychiatry. (2015) 49:898–905. 10.1177/0004867415585582 25991763

[46] Mur M Portella MJ Martínez-Arán A Pifarré J Vieta E. Long-term stability of cognitive impairment in bipolar disorder: a 2-year follow-up study of lithium-treated euthymic bipolar patients. J Clin Psychiatry. (2008) 69:712–9. 10.4088/JCP.v69n0504 18435565

[47] Baune BT Malhi GS. A review on the impact of cognitive dysfunction on social, occupational, and general functional outcomes in bipolar disorder. Bipolar Disord. (2015) 17 Suppl 2:41–55. 10.1111/bdi.12341 26688289

[48] Ryan KA Vederman AC Kamali M Marshall D Weldon AL McInnis MG. Emotion perception and executive functioning predict work status in euthymic bipolar disorder. Psychiatry Res. (2013) 210:472–8. 10.1016/j.psychres.2013.06.031 23870493

[49] MacQueen GM Memedovich KA. Cognitive dysfunction in major depression and bipolar disorder: assessment and treatment options. Psychiatry Clin Neurosci. (2017) 71:18–27. 10.1111/pcn.12463 27685435

[50] Sanchez-Moreno J Bonnin CM González-Pinto A Amann BL Solé B Balanzá-Martinez V. Factors associated with poor functional outcome in bipolar disorder: sociodemographic, clinical, and neurocognitive variables. Acta Psychiatr Scand. (2018) 138:145–54. 10.1111/acps.12894 29726004

[51] Hower H Lee EJ Jones RN Birmaher B Strober M Goldstein BI. Predictors of longitudinal psychosocial functioning in bipolar youth transitioning to adults. J Affect Disord. (2019) 246:578–85. 10.1016/j.jad.2018.12.108 30605876 PMC6363880

[52] Goldberg JF Allen MH Miklowitz DA Bowden CL Endick CJ Chessick CA. Suicidal ideation and pharmacotherapy among STEP-BD patients. Psychiatr Serv. (2005) 56:1534–40. 10.1176/appi.ps.56.12.1534 16339615

[53] Gazalle FK Frey BN Hallal PC Andreazza AC Cunha AB Santin A. Mismatch between self-reported quality of life and functional assessment in acute mania: a matter of unawareness of illness? J Affect Disord. (2007) 103:247–52. 10.1016/j.jad.2007.01.013 17289153

[54] Kamali M Reilly-Harrington NA Chang WC McInnis M McElroy SL Ketter TA. Bipolar depression and suicidal ideation: Moderators and mediators of a complex relationship. J Affect Disord. (2019) 259:164–72. 10.1016/j.jad.2019.08.032 31445343

[55] Kessler U Schoeyen HK Andreassen OA Eide GE Hammar Å Malt UF. Neurocognitive profiles in treatment-resistant bipolar I and bipolar II disorder depression. BMC Psychiatry. (2013) 13:105. 10.1186/1471-244X-13-105 23557429 PMC3637095

[56] Cullen B Ward J Graham NA Deary IJ Pell JP Smith DJ. Prevalence and correlates of cognitive impairment in euthymic adults with bipolar disorder: a systematic review. J Affect Disord. (2016) 205:165–81. 10.1016/j.jad.2016.06.063 27449549

[57] Gitlin MJ Miklowitz DJ. The difficult lives of individuals with bipolar disorder: a review of functional outcomes and their implications for treatment. J Affect Disord. (2017) 209:147–54. 10.1016/j.jad.2016.11.021 27914248 PMC7213058

[58] Santa Cruz EC Zandonadi FDS Fontes W Sussulini A. A pilot study indicating the dysregulation of the complement and coagulation cascades in treated schizophrenia and bipolar disorder patients. Biochim Biophys Acta Proteins Proteom. (2021) 1869:140657. 10.1016/j.bbapap.2021.140657 33839315

[59] Bonnin CM Martinez-Aran A Sanchez-Moreno J Torrent C Franco C Pacchiarotti I. [Bipolar disorder, cognitive functioning and hypothalamic-pituitary-thyroid axis]. Actas Esp Psiquiatr. (2010) 38:223–8. 21104467

[60] Chakrabarti S. Thyroid functions and bipolar affective disorder. J Thyroid Res. (2011) 2011:306–67. 10.4061/2011/306367 21808723 PMC3144691

[61] Levenberg K Hajnal A George DR Saunders EFH. Prolonged functional cerebral asymmetry as a consequence of dysfunctional parvocellular paraventricular hypothalamic nucleus signaling: an integrative model for the pathophysiology of bipolar disorder. Med Hypotheses. (2021) 146:110433. 10.1016/j.mehy.2020.110433 33317848

