# Supplementary figures and images for: Heterogeneity of psychosocial functioning in patients with bipolar disorder: Associations with sociodemographic, clinical, neurocognitive and biochemical variables
Source: Front Psychiatry. 2022 Sep 20;13:900757. doi: 10.3389/fpsyt.2022.900757 (PMC9530893; doi:10.3389/fpsyt.2022.900757)

&[ ]

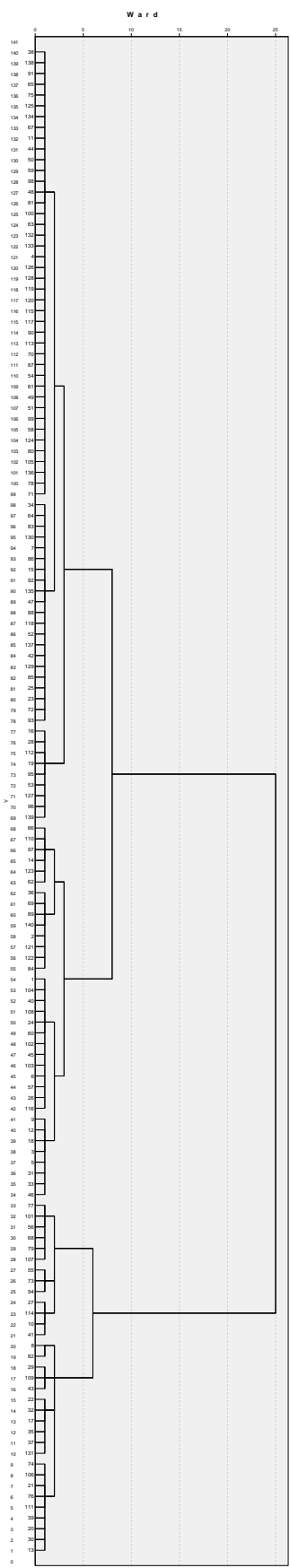

&[ ]

Supplement: Supplementary file 1 [file Data_Sheet_1.PDF]
